# VV-ECMO adjuvant therapy for *Leptospira* complicated with H1N1 infection: a case report

**DOI:** 10.3389/fmed.2024.1495324

**Published:** 2025-01-13

**Authors:** YuChou Zhang, MingJing Yin, HanChun Wen

**Affiliations:** Department of Critical Care Medicine, The First Affiliated Hospital of Guangxi Medical University, Guangxi Clinical Research Center for Critical Care Medicine, Guangxi, China

**Keywords:** leptospirosis, H1N1, VV-ECMO, ICU, penicillin, MNGs

## Abstract

**Background:**

Leptospirosis is an acute infectious disease that occurs by infection, progresses rapidly, and has a high mortality rate, with an estimated 1.2 million new cases and nearly 59,000 deaths each year. Due to its diverse clinical manifestations, diagnosis is often delayed. Therefore, it is necessary to pay attention to its clinical manifestations, diagnostic techniques, and treatment methods.

**Case report:**

A 51-year-old male patient from the Han ethnic group experienced fever, chills, headache, and overall fatigue after being exposed to rain, followed by yellowing of the skin and worsening of breathing difficulties. Metagenomic next-generation sequencing (mNGS) indicates infection with leptospirosis and influenza A. After 5 days of treatment with venovenous extracorporeal membrane oxygenation (VV-ECMO), Penicillium, and Oseltamivir, the condition improved.

**Conclusion:**

Leptospirosis improves with VV-ECMO support and anti-infective treatment with penicillin and oseltamivir. VV-ECMO provides a therapeutic time window for rescue, and mNGS lays a foundation for early detection of etiology of leptospirosis.

## Introduction

The global burden of leptospirosis as a tropical disease is greater than previously recognized, with an estimated 1.2 million new cases and nearly 59,000 deaths annually. Leptospirosis is widely acknowledged as one of the most geographically widespread zoonoses globally, yet underreporting likely hampers reliable global incidence estimates. Annually, over one million human cases, resulting in approximately 60,000 deaths, are reported worldwide (1). It is most common in tropical regions but can also occur in temperate regions (2). Human leptospirosis is typically sporadic, but outbreaks can occur from common exposure sources. Mammals serve as the primary reservoir of *Leptospira* bacteria. The environment can also act as a reservoir if contaminated with urine from infected mammals. *Leptospira* is transmitted to humans through portals of entry such as cuts, abrasions, mucous membranes, or the conjunctiva (3). In developing countries, leptospirosis causes severe illness and has become a significant cause of pulmonary hemorrhage and acute kidney failure, with 10% of patients dying and up to 70% bleeding.

Seasonal influenza is an acute respiratory infection caused by the influenza virus. Seasonal flu is common around the world. Most people recover. The World Health Organization (WHO) estimates that 5–10% of adults get flu each year. That includes 3 million to 5 million severe cases. Seasonal influenza causes 290,000 to 650,000 respiratory deaths each year (4). Symptoms begin 1–4 days after the infection and usually last about a week. For respiratory failure caused by influenza virus infection, extracorporeal membrane oxygenation (ECMO) should be considered.

ECMO is the use of a blood pump to generate power, establish a vascular pathway through venous catheterization, draw blood out, and then pass through the membrane lungs (5). After connecting to oxygen, the membrane lungs can diffuse oxygen into the blood and expel carbon dioxide through them. The membrane lungs convert venous blood into arterial blood. This arterial blood is then returned to the human body through the established perfusion pathway through vascular catheterization. The difference between venovenous ECMO (VV-ECMO) and venoarterial ECMO (VA-ECMO) lies in the selection of perfusion vessels. The perfusion vessel of VV-ECMO is the vein. The function of ECMO is to convert the venous blood in the right heart into arterial blood, replacing only the function of the lungs. The perfusion vessel of VA-ECMO is the artery. VA-ECMO is the process of extracting venous blood, converting it into arterial blood through the membrane lungs, and perfusing it into the arterial system. The substitute is cardiopulmonary function (6).

Tsutsugamushi disease is an acute natural infectious disease caused by the oriental body of tsutsugamushi disease. It is transmitted through the bite of mite larvae and is clinically characterized by fever, rash, characteristic scabs and ulcers, lymph nodes, and hepatosplenomegaly. It can cause complications such as toxic hepatitis, bronchopneumonia, myocarditis, meningoencephalitis, gastrointestinal bleeding, and acute renal failure. Tsutsugamushi disease is mainly distributed in Southeast Asia, with one million cases of jungle typhus occurring annually. The mortality rate for untreated or severe cases can be as high as 30% (7). Moreover, in the early stages of tsutsugamushi disease, specific symptom manifestations and diagnostic indicators are absent, coupled with insufficient understanding by clinical doctors, making it highly susceptible to misdiagnosis and missed diagnosis.

Catheter-related blood flow refers to the occurrence of bacteremia or mycosis in patients with intravascular catheters or those who have had their catheters removed within 48 h, accompanied by symptoms of infection such as fever (>38°C), chills, or hypotension, without any clear source of infection other than vascular catheters. Common bacterial infections include coagulase negative *Staphylococcus aureus*, which account for approximately 50% of pathogenic bacteria (8).

In this article, the use of VV-ECMO in combination with penicillin and oseltamivir for the treatment of leptospirosis with swine flu (H1N1) infection is reported.

## Case

**Figure 1:**
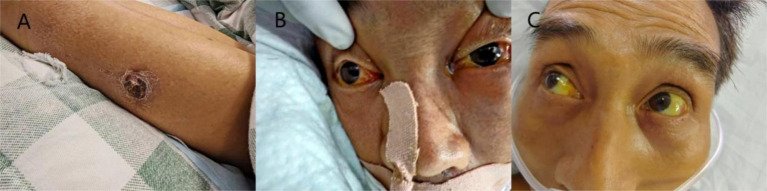
**(A)** Physical examination showed a 2 × 2 cm crusted wound on the outside of the left calf. **(B)** On June 14, it was observed that the patient had conjunctival congestion. **(C)** On June 25, after treatment with penicillin, the patient’s conjunctival congestion subsided.

**Figure 2:**
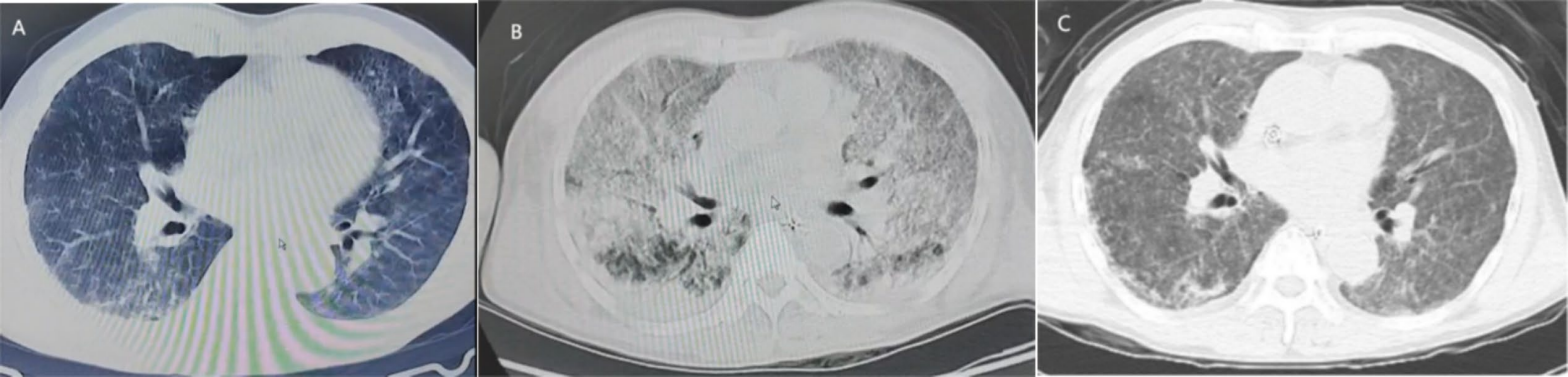
The image shows the patient’s lung condition and changes after treatment. **(A)** On June 11, the patient’s lung CT showed slight exudative lesions in both lungs. **(B)** On June 13, pulmonary CT showed diffuse exudative lesions in both lungs. **(C)** On June 17, lung CT showed significant improvement after treatment.

The patient is a 51-year-old Han man who was admitted to the hospital due to fever, fatigue for 9 days, and progressive respiratory distress for 8 days. The patient developed fever, chills, headache, and overall weakness after being caught in the rain while fishing in the wild on June 6, 2024. On June 7, 2024, the patient experienced breathing difficulties accompanied by nausea and vomiting, and the breathing difficulties gradually worsened. On June 11, 2024, a positive case of influenza A virus was found in the local hospital. Physical examination revealed a 2 × 2 cm scab wound on the outer side of the left calf (Figure 1). Yellow staining throughout the body, wet rales covering both lungs, and diffuse exudative lesions visible on computed tomography (CT) scans of both lungs (Figure 2A) were observed. Laboratory examination showed the following values: total bilirubin, 261umol/L; albumin, 17 g/L; alanine transaminase (ALT), 36 U/L; aspartate transaminase (AST), 75 U/L; erythrocyte sedimentation rate (ESR), 86 mm/h; urine protein(+); creatinine, 606umol/L, urea nitrogen was 35 mmol/L. At the time of diagnosis, it was suspected that tsutsugamushi disease was complicated with H1N1 infection. On June 13, 2024, tracheal intubation and ventilator-assisted respiration were performed, using meropenem, doxycycline, voriconazole for anti-infection, vasopressor drugs, continuous renal replacement therapy (CRRT), and symptomatic treatment. A re-examination of CT showed a significant increase in diffuse exudative lesions in both lungs compared to before (Figure 2B). After treatment, the patient’s oxygen partial pressure is still low at 100% oxygen concentration, with blood pressure of 83/58 mmHg and oxygenation index of 33 mmHg. On June 14, 2024, the patient underwent VV-ECMO treatment and was transferred to the Intensive Care Department of the First Affiliated Hospital of Guangxi Medical University on June 14, 2024. At admission, the following readings were observed: body temperature, 35.8°C; pulse, 56 beats/min; respiration, 14 beats/min; blood pressure, 138/69 mmHg (0.2 ug/kg/min norepinephrine), and conjunctival congestion (Figure 1A).

**Figure 3:**
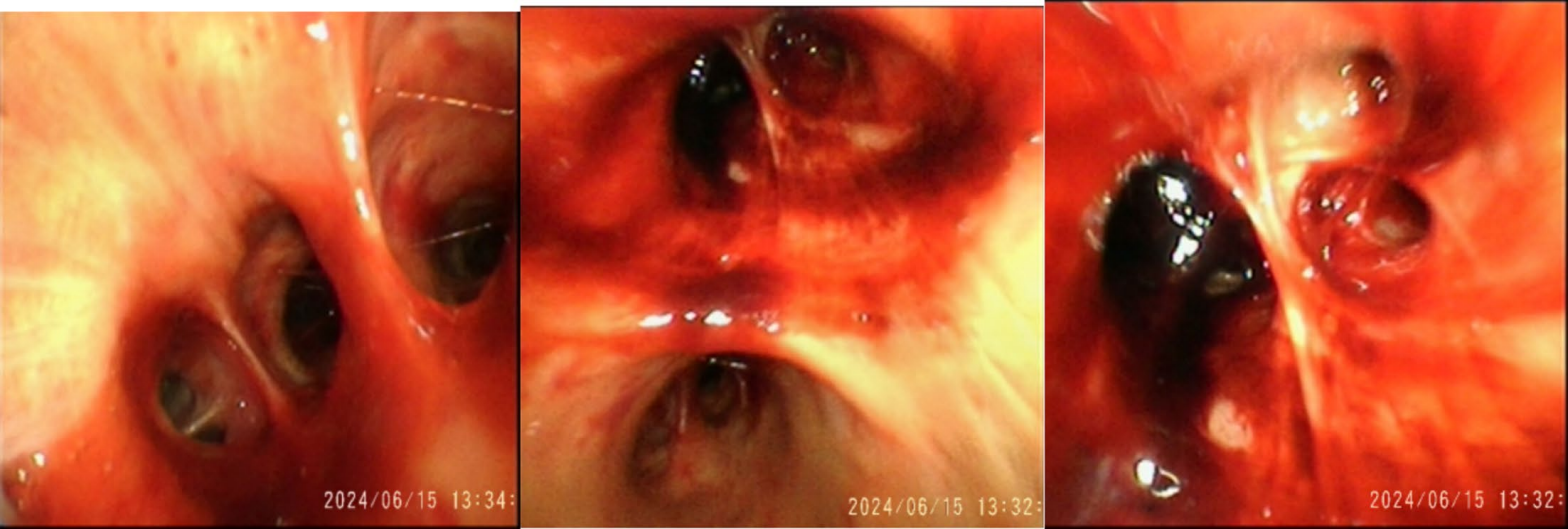
On June 15, 2024, diffuse bloody secretions were observed under fiberoptic bronchoscopy.

No lymph node enlargement was observed. The VV-ECMO speed is 2,800 r/min, blood flow rate is 3.5 L/min, and gas flow rate is 3 L/min. The anti-infection regimen is oseltamivir and imipenem cilastatin sodium. On June 15, 2024, diffuse bloody secretions of the airway were observed under fiberoptic bronchoscopy (Figure 3), and metagenomic next-generation sequencing (mNGS) was performed on the patient’s blood and bronchoalveolar lavage fluid. The mNGS of blood only indicates a possibility of *Leptospira* infection (blood reads 2). No abnormalities were found in the mNGS of alveolar lavage fluid. After the pathogen was identified, we used penicillin (1.6 million units q6h) to combat the infection.

**Figure 4:**
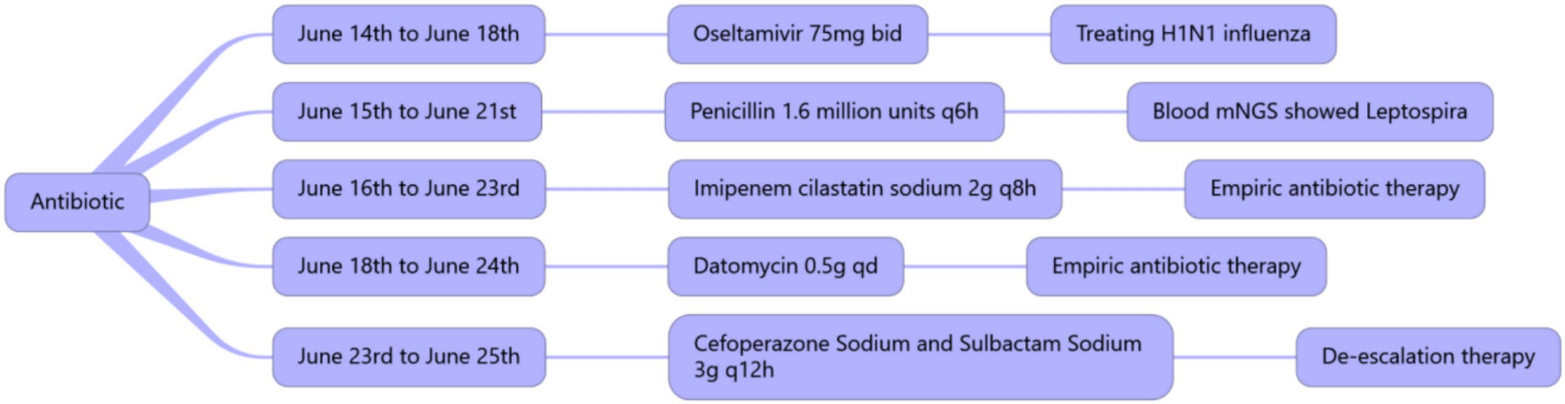
Summary of the usage, dosage, and duration of antibiotics.

On June 17, 2024, a high fever occurred with a maximum body temperature of 38.7°C. White blood cells gradually increased, reaching a maximum of 35.3 × 10^9^/L, suggesting the possibility of catheter-related bloodstream infection. Datomycin was added to treat the infection. After treatment, the patient’s vital signs remained stable. Figure 4 summarizes the antibiotic therapy. On June 18, 2024, the condition improved and the patient was evacuated from VV-ECMO. On June 17, 2024, there was a significant improvement in the lung CT after re-examination (Figure 2C).

The ventilator was discontinued on June 20, 2024, and the tracheal tube was removed on June 22, 2024.

## Discussion

A review of the patient’s case showed that at the beginning, the patient had an unknown cause of fever, a history of fishing in the field, and a 2 × 2 cm crusted wound on the outside of the left calf. Tsutsugamushi disease was considered at that time, and the symptoms of dyspnea progressed rapidly. The clinical diagnosis of leptospirosis is relatively difficult, and non-specific symptoms include elevated white blood cells, positive urine protein, thrombocytopenia in critically ill patients, and left shift of neutrophil nuclei in peripheral blood (9). The patient in the case met the first two criteria. Specific tests include microaggregation test, enzyme-linked immunosorbent assay, and other tests. However, many hospitals lack instruments for detecting relevant indicators. In summary, the diagnosis of leptospirosis is relatively difficult (9).

However, the etiology of the patient was identified by mNGS results, and the patient quickly improved after symptomatic treatment. After reviewing the patient’s history, we considered that the patient was infected with leptospirosis after contact with a water source containing leptospirosis following a left calf injury. By reviewing the literature reports, we found that the peak time of tsutsugamushi disease in southern China was from June to September (10), which was consistent with that of leptospirosis in southern China. Moreover, tsutsugamushi disease and leptospirosis were similar in terms of infection route, symptoms, and signs. The difference was that leptospirosis would show congestion of ocular conjunctival, gastrocnemius pain and lymph node enlargement about 3 days in the early stage of the disease. In the middle stage of leptospirosis at about 3–10 days, organ function damage will appear, and according to the degree of damage to the organ, it can be divided into ordinary type, pulmonary hemorrhage, jaundice hemorrhage, kidney failure type, and meningoencephalitis type. This case had diffuse bloody secretions in the lungs and visible damage to liver and kidney function, suggesting jaundice hemorrhagic leptospirosis. It can be seen that patients infected with leptospirosis have sustained kidney and liver damage but have not developed more serious complications such as hepatic encephalopathy and more severe kidney failure. For the treatment of leptospirosis, there have been no reports of penicillin resistance. *In vitro* studies and animal models have shown that penicillins, cephalosporins, tetracycline, chloramphenicol, fluoroquinolones, macroliones, and telithromycin are effective against leptospirosis. In vitro studies have shown that carbapenems and amtronam are also very effective against leptospirosis (11–13).

After influenza A (H1N1) virus inhalation through the respiratory tract, the hemagglutinin (HA) on the virus surface recognizes and binds to the host cell surface receptor, infects the ciliated columnar epithelial cells of the respiratory tract, replicates in the cell, and releases the virus from the cell with the help of neuraminidase (NA), and then infects other fibrous columnar epithelial cells, causing cell degeneration, necrosis and fall-off, resulting in local inflammation. Then systemic toxic reactions occur (14). At the same time, the virus can also invade the trachea and bronchus down to the alveoli, resulting in influenza viral pneumonia. In this case, the patient was infected with influenza A (H1N1) virus combined with leptospirosis, and the lung CT showed diffuse exuding lesions in both lungs, which progressed rapidly and led to respiratory failure. Therefore, VV-ECMO treatment is required. VV-ECMO technology drains venous blood from patients to the outside of the body, and after oxygenation and carbon dioxide removal, it is returned to the patient to undertake gas exchange and/or part of the blood circulation function. VV-ECMO is only respiratory aid. In this case, VV-ECMO support is also a key factor in the success of treatment.

## Conclusion

This study describes a case of leptospirosis with influenza A (H1N1) virus infection confirmed by mNGS. The patient’s condition improved with VV-ECMO support and anti-infective treatment with penicillin and oseltamivir. VV-ECMO provides a therapeutic time window for rescue, and mNGS lays a foundation for early detection of etiology.

## Data Availability

The original contributions presented in the study are included in the article/supplementary material, further inquiries can be directed to the corresponding author.
